# Thyroid autoimmunity and its negative impact on female fertility and maternal pregnancy outcomes

**DOI:** 10.3389/fendo.2022.1049665

**Published:** 2023-01-11

**Authors:** Kamila Tańska, Małgorzata Gietka-Czernel, Piotr Glinicki, Jarosław Kozakowski

**Affiliations:** Department of Endocrinology, Centre of Postgraduate Medical Education, Warsaw, Poland

**Keywords:** thyroid autoimmunity, female fertility, pregnancy outcomes, assisted reproductive technology outcomes, levothyroxine treatment, immunotherapy

## Abstract

Thyroid autoimmunity (TAI) is commonly defined as the presence of thyroperoxidase antibodies (TPOAbs) and/or thyroglobulin antibodies (TgAbs), which predisposes an individual to hypothyroidism. TAI affects nearly 10% of women of reproductive age and evokes great interest from clinicians because of its potentially negative impact on female fertility and pregnancy course. In this mini-review, we review the current literature concerning the influence of TPOAb or TPOAb/TgAb positivity without thyroid dysfunction on reproduction. TAI may negatively affect female fertility; several studies have found an increased prevalence of TAI in infertile women, especially in those with unexplained infertility and polycystic ovary syndrome. According to some observations, TAI might also be connected with premature ovarian insufficiency and endometriosis. The relationship between TAI and an increased risk of pregnancy loss is well documented. The pathophysiological background of these observations remains unclear, and researchers hypothesize on the direct infiltration of reproductive organs by thyroid antibodies, co-existence of TAI with other autoimmune diseases (either organ specific or systemic), immunological dysfunction leading to inhibition of immune tolerance, and relative thyroid hormone deficiency. Interestingly, in the current literature, better outcomes of assisted reproductive technology in women with TAI have been reported compared with those reported in earlier publications. One plausible explanation is the more widespread use of the intracytoplasmic sperm injection method. The results of randomized clinical trials have shown that levothyroxine supplementation is ineffective in preventing adverse pregnancy outcomes in women with TAI, and future research should probably be directed toward immunotherapy.

## Introduction

Thyroid autoimmunity (TAI) is commonly characterized by the presence of circulating thyroperoxidase antibodies (TPOAbs) and/or thyroglobulin antibodies (TgAbs) which predisposes to hypothyroidism. In this mini-review, we will focus on the consequences of TAI with elevated concentrations of TPOAb and/or TgAb without thyroid dysfunction for female fertility and maternal obstetric complications.

TAI is the most common autoimmune disease among women of reproductive age (1). Recent publications report the presence of TPOAbs and TgAbs in 5%–14% and 3%–18%, respectively, of pregnant women (2). Moreover, TPOAbs were found in 9.5% of women with previous pregnancy loss or subfertility (3). TPOAb positivity appeared to be connected with iodine deficiency or excess (4), age over 40 years (5), obesity classes II–III (body mass index ≥35 kg/m^2^), and ethnicity, occurring more frequently in Caucasian women than in Black women (3). TPOAb prevalence in pregnant women might be underestimated because the currently used manufacturer cutoffs for TPOAb may be too high for this population. In a recent study, TPOAb concentrations below the manufacturer cutoffs were associated with higher thyroid-stimulating hormone (TSH) concentrations and a higher risk of premature delivery (6). Due to the fact that TPOAb is recognized as a sensitive marker of TAI, which is associated with the risk of hypothyroidism (7), the majority of publications explore the impact of TPOAb or TPOAb/TgAb, while there are few studies investigating the effect of isolated TgAb. However, isolated TgAb positivity may be as common as isolated TPOAb positivity: 5% vs. 4% (8) and 14.5% vs. 13.5% (5), respectively, and may exert some harmful effects. So far, it has been demonstrated that TgAb can interfere with the thyroidal response to human chorionic gonadotropin (hCG) stimulation similar to TPOAb (9). TgAbs are associated with an increased risk of premature rupture of fetal membranes and low birth weight (10). They might also have a significant impact on TSH concentration (11). In its 2017 guidelines, the American Thyroid Association (ATA) stated that there is a need for further research on the significance of isolated TgAb positivity (12). Increasing evidence confirms the association between TAI and the risk of pregnancy loss and suggests its relationship with reduced fertility. Unfortunately, the pathological pathways through which TAI exerts its adverse effects remain unclear; therefore, treatment options are limited.

## Pathophysiology

The association between TAI and procreation can be direct or indirect. A hypothesis on a probable direct harmful effect of thyroid antibodies comes from a small Italian study in which a group of 14 infertile women with TAI undergoing *in vitro* fertilization (IVF) had TPOAbs and TgAbs in the follicular fluid, where their concentrations correlated positively with those in the blood. Oocyte fertilization, high-quality embryos, and pregnancy rates were lower in TAI women than in 17 negative controls (13). These results were then replicated in a larger study group (14), which suggested that TPO is a common antigen in both thyroid and ovarian tissues (15). As TPOAb has been proven to cause thyrocyte death through antibody-dependent cytotoxicity cells (ADCC) and C3 complement-mediated cytotoxicity and TgAb has been postulated to act *via* ADCC (16), damage to reproductive organs expressing TPO and Tg might ensue. Additionally, the cross-reactivity of TPOAb with hCG receptors in the zona pellucida has been proposed as another potential mechanism of infertility (17).

As all the components necessary to produce thyroid hormones are present in the endometrium, syncytiotrophoblast, and invasive trophoblast (sodium/iodide symporter, pendrin, TPO, and Tg), one can speculate about the local synthesis of thyroid hormones. In such circumstances, thyroid autoimmunity could lead to thyroid hormone deficiency at the tissue level and disturb the process of embryo implantation and placentation, with consequent infertility or obstetrical complications (1, 18, 19). To support this hypothesis, according to two publications by Italian researchers (20, 21), TAI was linked to increased uterine artery resistance and umbilical artery vasoconstriction, as determined by the Doppler technique. Hemodynamic abnormalities were associated with increased rates of miscarriage, fetal growth restriction, small for gestational age, preeclampsia, and prematurity.

The indirect negative impact of TAI on fertility and pregnancy outcomes might be its co-occurrence with other autoimmune diseases, either organ specific (autoimmune polyglandular syndromes 1–4) with anti-ovarian antibodies or systemic (systemic lupus erythematosus, Sjögren’s syndrome, and rheumatoid arthritis) with antinuclear antibodies, antiphospholipid antibodies, and anti-laminin-1 antibodies (aLN-1), which have documented negative impacts on fertility and pregnancy outcomes (22, 23). It has been reported that 20% of women with TAI have non-organ-specific antibodies that can react with the trophoblast/placenta and induce a prothrombotic state, cytokine imbalance, and complement activation (17).

TAI itself can lead to immunological dysfunction and inhibition of immune tolerance at the systemic and maternal–fetal interface levels. A systemic imbalance of helper lymphocyte Th1/Th2/Th17 and Treg activity, leading to increased secretion of inflammatory cytokines interleukin (Il)-2, IL-17, and interferon-γ (INF-γ)—known factors of implantation failure and pregnancy loss—was observed in women with TAI (24, 25). In addition, excessive activation and cytotoxicity of natural killer (NK) cells in peripheral blood and the uterus, as well as upregulation of NKT-like cells, have been reported (26–28). Interestingly, NK cell numbers and activity can be enhanced not only by Il-2 and INF-γ but also by TSH acting in an endocrine and paracrine manner (17).

Another potentially negative factor in women with TAI might be a relative deficiency of thyroid hormone. Several studies have found that thyroid autoimmunity is associated with higher serum TSH levels within the normal range compared with healthy controls (3, 6, 29). In addition, about 20% of TAI women who were euthyroid in the preconception period developed subclinical hypothyroidism over the course of their pregnancies. In a study by Korevaar et al. (30), TPOAb positivity was associated with an impaired thyroidal response to hCG during pregnancy. TPOAb-positive women with a more-impaired-than-expected thyroidal response to hCG (lower FT4 than expected for hCG) had more than 2.0-fold higher risk of premature delivery. Furthermore, the risk of a negative pregnancy outcome can be modified by TSH concentration, and the combination of TPOAb positivity with high-normal TSH concentration is associated with synergistically higher risks of miscarriage (31) and preterm delivery (6).

Previous publications have pointed out that the older age of women with TAI is an independent factor for decreased fertility and a higher risk of miscarriage (32). However, in a recent publication, infertile women with TAI were not older than women without thyroid autoimmunity (33).

## Thyroid autoimmunity and fertility

Publications assessing the prevalence of thyroid autoantibodies in infertile women have reported divergent results, which may be explained by heterogeneous study designs (retrospective, prospective, and cross-sectional), different populations studied (various ages of subjects and causes of infertility), and different assays used to determine thyroid antibodies. It is noteworthy that, in a recent prospective study carried out among 1,054 fertile women with a history of one or two prior pregnancy losses, there was no difference in the pregnancy rates between 154 women with TAI and 900 women without TAI (74% vs. 72.2%, respectively, *p* = 0.64) (34). However, summarized data have shown an association between TAI and decreased fertility. In a review study of women with various causes of infertility, Poppe et al. demonstrated a significantly higher incidence of TAI [risk ratio (RR): 2.1, *p <* 0.0001] (35). In a meta-analysis by van den Boogaard et al. (36), the presence of thyroid antibodies was associated with a higher risk of unexplained subfertility [odds ratio (OR): 1.5, 95% confidence interval (95% CI): 1.1–2.0]. An elevated prevalence of TAI might be especially concerning in some particular causes of infertility: polycystic ovary syndrome (PCOS) (26.9%), idiopathic infertility, and endometriosis (25%) (37–40). In a 2018 meta-analysis of 13 cross-sectional and case–control studies evaluating a total of 1,210 women with PCOS and 987 healthy controls, Romitti et al. (41) found a significant association between PCOS and TAI (OR: 3.27, 95% CI: 2.32–4.63). A predisposing factor for the co-occurrence of PCOS and TAI is a polymorphism in the fibrillin gene, which regulates transforming growth factor-β (TGF-β) activity, which, in turn, affects Treg cells. Reduced TGF-β and Treg activity promotes the development of autoimmune diseases. Another predisposing factor is the high estrogen/progesterone ratio found in women with PCOS and vitamin D deficiency (42). TAI leading to (sub)hypothyroidism may negatively affect metabolic performance, higher triglycerides, and free testosterone in women with PCOS (43). Some studies have found TAI in 25%–46% of women with endometriosis attending infertility clinics (29, 40, 44), although this observation has not been confirmed by other observations (41, 45). The pathophysiology of endometriosis is complex and still unclear, but the condition is associated with a variety of inflammatory and immunological phenomena, such as the presence of autoantibodies to endometrial antigens (including aLN-1), complement deposits, apoptosis, a decline in NK cell concentration, and cytotoxic effects on the endometrium (46, 47). Reciprocally, thyroid antibodies can affect the human endometrium, including ectopic endometrium, as all of the transcripts involved in thyroxin synthesis have been found in the endometrium, including TPO and Tg (1). Several studies have pointed out a possible association between TAI and diminished ovarian reserve or premature ovarian insufficiency (POI). From 4% to 30% of POI cases are autoimmune in origin (23); in a recent meta-analysis, the authors confirmed a higher frequency of TPOAb positivity in this group of patients (OR: 2.26, 95% CI: 1.31–3.92, *p* = 0.004), but not of TgAb positivity (48). After pooling data from 30 studies published between 1997 and 2021, they concluded that women of reproductive age with Hashimoto’s thyroiditis (hypothyroid and euthyroid) have lower concentrations of anti-Müllerian hormone and antral follicle count. Unfortunately, they did not perform a subanalysis in a group of euthyroid women. In a recent publication (49) not included in the abovementioned meta-analysis, retrospective research of 4,302 euthyroid women proved that TAI was associated with POI only in the group with TSH > 2.5 µIU/ml but not in those with TSH ≤ 2.5 µIU/ml. These facts indicate that there may be a role for relative thyroid hormone insufficiency acting together with thyroid autoimmunity in the process of ovarian damage. The European Society of Human Reproduction and Embryology recommends testing for TPOAb in women with POI (50).

## Thyroid autoimmunity and the risk of maternal obstetric complications

The association between TAI and miscarriage is well documented. In a 2011 meta-analysis of 31 studies comprising 12,126 women without overt thyroid dysfunction, an elevated risk of miscarriage was demonstrated among women with TPOAb/TgAb positivity (OR: 3.90, 95% CI: 2.48–6.12, *p* < 0.001) (51). This association was also proven among euthyroid women with thyroid antibody positivity (OR: 1.80, 95% CI: 1.25–2.60, *p* = 0.002) but was only confirmed in two subgroups: women with recurrent miscarriage and women with infertility. A significant doubling in the odds of preterm birth with the presence of thyroid autoantibodies was also demonstrated (OR: 2.07, 95% CI: 1.17–3.68, *p* = 0.01). An elevated risk of preterm delivery was documented in another meta-analysis comprising 11 prospective cohort studies and 35,467 participants; the relative risk of preterm delivery was higher for pregnant women with thyroid antibodies compared with controls (RR: 1.41, 95% CI: 1.08–1.84, *p* = 0.011) and for TPOAb-positive euthyroid women (RR: 1.98, 95% CI: 1.29–3.04, p = 0.002), but not for TgAb positivity (52). The latest meta-analysis of 19 cohorts pooling the data of 47,055 pregnant women also demonstrated that TPOAb-positive euthyroid women had a higher risk of preterm birth vs. TPOAb-negative women (6.8% vs. 4.9%) (OR: 1.36, 95% CI: 1.15–1.60, *p* < 0.01) (53). Three current studies, two prospective (54, 55) and one retrospective (56), have confirmed the previously established relationship between TAI and the risk of miscarriage, preterm birth, and early-term birth. It is interesting that, in one of the studies, the association between TAI and preterm birth occurred only in cases with female fetuses (55).

The association between TAI and recurrent pregnancy loss was demonstrated in a recent meta-analysis (57). After pooling data from 17 studies that included women with TPOAb positivity or TPOAb/TgAb positivity, the meta-analysis revealed a statistically significant association between recurrent pregnancy loss and thyroid autoimmunity (OR: 1.94, 95% CI: 1.43–2.64).

In a recent study of 454 women with unexplained recurrent pregnancy loss, TPOAb positivity was associated with a lower live birth rate (51.3% vs. 65.2%, *p* = 0.02) (58). However, the conclusion is hindered because 75% of the TPOAb-positive women and 3.7% of the TPOAb-negative women received L-thyroxine treatment.

Several studies, mostly retrospective, have reported an increased risk of preeclampsia, gestational diabetes mellitus (GDM), anemia, placenta previa, polyhydramnios, placental abruption, and premature rupture of membranes in women with TAI (59). Although these observations are inconsistent and further studies are needed, the last complication has been documented in a cohort of 10,062 women (60, 61). It is interesting that placental abruption was linked to the persistence of TPOAb positivity in the first and second trimesters, and the risk was doubled when TPOAb together with TgAb was increased. It should be noted that the link between TAI and GDM requires further investigation, but it was suggested in the latest meta-analysis (62). The authors found an increased risk of GDM in TAI pregnant women with TSH <4.0 mIU/L, while there was no such risk in controls with TSH <4.0 mIU/L and negative thyroid antibodies (OR: 2.04, 95% CI: 1.32–3.137, *p* < 0.001). The plausible link between TAI and GDM is an elevated concentration of inflammatory cytokines, which leads to insulin resistance (63).

## Thyroid autoimmunity and assisted reproductive technology outcomes

The impact of thyroid autoimmunity on assisted reproductive technology (ART) outcomes has been widely investigated, but the studies have great heterogeneity due to different designs, causes of infertility, various protocols for ovarian stimulation, various definitions of euthyroidism, different fertilization procedures, including IVF, intracytoplasmic sperm injection (ICSI), or intrauterine insemination (IUI), and variously defined outcomes. Previous studies have reported poorer embryo quality (64, 65), lower clinical pregnancy rates, higher risk of miscarriage, and lower live birth rates in women with TAI undergoing ART (66–68). However, in several recent studies (69–71), comprehensive reviews (72, 73), and meta-analyses (74–76), no deleterious effect of TAI on ART outcomes was found. The suggested explanation for this discrepancy might be the increasing usage of the ICSI method of fertilization. As this procedure involves injecting sperm into the center of the egg, it overcomes the potential barrier of thyroid antibodies infiltrating the zona pellucida. However, this hypothesis does not explain the lack of a negative relationship between TAI and IUI outcomes observed at present (77). Although ICSI has mainly been performed in male infertility, this method is suggested by the European Thyroid Association (ETA) to be used in infertile women with thyroid autoimmunity (78).

Special attention should be paid to the risk of hypothyroidism in women with TAI undergoing ART. Diminished thyroid response to hCG, on the one hand, and rapid increase of estradiol and thyroxin-binding globulin concentrations soon after controlled ovarian stimulation, on the other hand, might result in a decrease in free thyroid hormone accessibility (79, 80). Monitoring thyroid function in women with TAI undergoing ART was proposed in the 2021 ETA guidelines (78) and adopted by some endocrine societies (81). It consists of assessing the TSH concentration at the time of the second positive hCG result confirming pregnancy.

An important question exists: “What is the optimal preconceptional value of TSH concentration determining successful ART outcomes in women with TAI?” Unaune et al. did not observe significant differences in cumulative delivery rates after IVF/ICSI between TPOAb-positive and TPOAb-negative women whenever the TSH threshold of 2.5 or 5.0 mIU/L was adopted (69). Similarly, Chai et al. (82) found no difference in IVF outcomes between women with TSH below and above 4.5 mIU/L. Furthermore, in a recent meta-analysis including 18 publications and 14,846 participants, no difference was observed in IVF/ICSI/IUI outcomes when a TSH cutoff value of 2.5 mIU/L was used (83). However, when a broader TSH cutoff value of 3.5–5 mIU/L was used, a higher miscarriage rate was observed (RR: 1.91, 95% CI: 1.09–3.35, *p* = 0.02). Unfortunately, thyroid autoimmunity status was not taken into account in the studies’ selection criteria.

## Treatment options

### Levothyroxine

According to the hypothesis postulating that TAI is accompanied by a relative thyroid hormone deficiency in the blood and/or at the tissue level, supplementation with levothyroxine may have beneficial effects on pregnancy outcome. The 2017 ATA guidelines (12) recommended levothyroxine treatment for women with thyroid autoimmunity and TSH above the pregnancy-specific reference range. The ATA also proposed considering levothyroxine for women with thyroid autoimmunity and TSH above 2.5 mIU/L and for euthyroid infertile women with a history of pregnancy loss. After the publication of the ATA guidelines, several important randomized clinical trials (RCTs) were released, which makes it necessary to reconsider this perspective (**Table 1**) (84–90). Moreover, four meta-analyses including only RCTs could not find any evidence that levothyroxine supplementation in euthyroid women with AITD resulted in an improvement in maternal pregnancy outcomes (94–97). The results of the meta-analyses have also shown that initiating levothyroxine during the preconception period or in the first trimester did not affect the miscarriage risk. Finally, the highly anticipated T4-LIFE trial addressing the impact of levothyroxine treatment in TPOAb-positive euthyroid women with recurrent miscarriage was published, and the levothyroxine intervention failed again to decrease the risk of pregnancy loss (90). Although these observations require further analysis, the only beneficial effect of levothyroxine treatment in TAI women documented so far is prevention of hypothyroidism.

**Table 1:** Prospective randomized clinical studies of levothyroxine treatment and selenium supplementation in euthyroid pregnant women with thyroid autoimmunity.

Trial	Study groups/type of pregnancy	Type of antithyroid antibody	Intervention	Main results
Levothyroxine treatment				
Negro et al. 2005, Italy (84)	Treated group *n* = 36 Placebo group *n* = 36	TPOAb	LT4 instituted before ART at a dose of 1 µg/kg/day and maintained throughout pregnancy	Significant reduction in miscarriage rates in LT4 group vs. placebo: 33% vs. 52%, *p* = 0.028
	Type of pregnancy: ART			
Negro et al. 2006, Italy (85)	Treated group *n* = 57 Untreated group *n* = 58	TPOAb	LT4 started at a mean 10 weeks of gestation TSH <1.0 mIU/L-LT4 dose of 0.5 µg/kg/day TSH between 1.0 and 2.0 mIU/L-LT4 dose of 0.75 µg/kg/day TSH >2.0 mIU/L or TPOAb >1,500 kIU/L-LT4 dose of 1 µg/kg/day	Significant decrease in the rates of miscarriage and preterm birth in LT4 group vs. controls: 3.5% vs. 13.8%, *p* < 0.05 and 7% vs. 22.4%, *p* < 0.05
	Type of pregnancy: spontaneous			
Negro et al. 2016, Italy (86)	Treated group *n* = 198 Untreated group *n* = 195	TPOAb	LT4 instituted at the first trimester of pregnancy TSH between 0.5 and 1.5 mIU/L-LT4 dose of 0.5 µg/kg/day TSH between 1.5 and 2.5 mIU/L-LT4 dose of 1 µg/kg/day	LT4 intervention with no significant impact on the rates of: miscarriage: 11.6% vs. 14.9%, *p* = 0.11 and preterm birth: 6.9% vs.10.8%, *p* = 0.27
	Type of pregnancy: spontaneous			
Wang et al. 2017, China (87)	Treated group *n* = 300 Untreated group *n* = 300	TPOAb	Starting dose at preconception period: TSH <2.5 mIU/ ml- LT4 dose of 25 µg/day TSH ≥2.5 mIU/ml- LT4 dose of 50 µg/day During pregnancy, the LT4 dose was titrated to maintain the TSH level within 0.1–2.5 mIU/L in the first trimester, 0.2–3.0 mIU/L in the second trimester, and 0.3–3.0 mIU/L in the third trimester	No difference between LT4 group and controls in the rates of: live birth: 31.7% vs. 32.3% pregnancy: 35.7% vs. 37.7% miscarriage: 10.3% vs. 10.6%
	Type of pregnancy: ART			
Nazarpour et al. 2017, Iran (88)	Treated group *n* = 18^a^ Untreated group *n* = 24^a^	TPOAb	LT4 instituted at a mean of 11 weeks of gestation TSH <1.0 mIU/L- LT4 dose of 0.5 µg/kg/day TSH between 1.0 and 2.0 mIU/L– LT4 dose of 0.75 µg/kg/day TSH >2.0 mIU/L or TPOAb >1,500 kIU/L- LT4 dose of 1 µg/kg/day	No difference between LT4 and control groups in the rates of preterm birth: 11.1% vs. 16.7%, *p* = 0.69
	Type of pregnancy: spontaneous			
Dhillon-Smith et al. 2019, United Kingdom (89)	Treated group *n* = 476 Placebo group *n* = 476 Women with history of miscarriage and/or infertility	TPOAb	LT4 instituted before conception at a fixed dose of 50 µg/day and maintained throughout pregnancy	No difference between LT4 and placebo groups in the rates of: live birth 37.4% vs. 37.9% pregnancy 56.6% vs. 58.3% miscarriage 28.2% vs. 29.6%
	Type of pregnancy: spontaneous or ART			
Van Dijk et al. 2022, Netherlands (90)	Treated group *n* = 94 Placebo group *n* = 93 Women with recurrent pregnancy loss	TPOAb	LT4 instituted before conception and continued at the same dose throughout pregnancy TSH <1.0 mIU/L- LT4 dose of 0.5 µg/kg/day TSH between 1.0 and 2.5 mIU/L- LT4 dose of 0.75 µg/kg/day TSH>2.5 mIU/L- LT4 dose of 1 µg/kg/day	LT4 treatment compared with placebo, did not result in higher live birth rates: 50% vs. 48%
	Type of pregnancy: spontaneous or ART			
Selenium supplementation				
Negro et al. 2007, Italy (91)	Treated group *n* = 77 Placebo group *n* = 74	TPOAb	Selenomethionine 200 µg/day or placebo from 12 weeks of gestation until delivery and 12 months postpartum	In the Se-treated group, the incidence of postpartum thyroiditis and permanent hypothyroidism after delivery were significantly lower compared with placebo group. Se therapy decreased the TPOAb concentration in postpartum period but did not influence TSH and fT4 during pregnancy and maternal/fetal complications 19.4% in Se group and 21.6% in placebo group required LT4 treatment during pregnancy
	Type of pregnancy: spontaneous			
Mao et al. 2016, United Kingdom (92)	Treated group *n* = 10 Placebo group *n* = 15	TPOAb/TgAb	Selenium-enriched yeast 60 µg/day or placebo from 12 weeks of gestation until delivery	Among TPOAb/TgAb-positive pregnant women, TSH and fT4 dropped significantly lower in the Se-treated group than in the placebo group. Se did not influence the TPOAb concentration
	Type of pregnancy: spontaneous			
Mantovani et al. 2019, Italy (93)	Treated group *n* = 21 Placebo group *n* = 24	TPOAb/TgAb	Selenomethionine 83 µg/day or placebo from 10 weeks of gestation until delivery and 6 months postpartum	There was no effect of Se on TPOAb and TgAb during pregnancy in comparison to placebo group After delivery, a significant reduction of TPOAb and TgAb was noted in the Se group, while antibody titers rebounded in the placebo group No differences were found in thyroid function, volume, echogenicity, quality of life, maternal/fetal complications between Se, and placebo groups 56.5% in Se group and 70% in the placebo group required LT4 treatment during pregnancy
	Type of pregnancy: spontaneous			

a only a subgroup of women with euthyroidism defined as TSH< 4.0 is presented; LT4, levothyroxine; ART, assisted reproductive technology; TPOAb, thyroperoxidase antibody; TgAb, thyroglobulin antibody; TSH, thyroid stimulating hormone; Se, selenium.

### Selenium

Selenium (Se) is a micronutrient with immunoregulatory properties and a well-established role in thyroid physiology (98, 99). In many previous studies, Se supplementation reduced the TPOAb and TgAb levels and improved the thyroid echogenicity (100–104). Although Se deficiency has been reported in several European countries, especially among pregnant women (105, 106), the efficacy of Se supplementation among pregnant women with TAI remains controversial (**Table 1**) (91–93). In the future, many aspects of Se therapy should be taken into account, such as its interaction with other micronutrients, especially iodine, and the postulated inverted U-shape relationship with disease, which means that either Se deficiency or Se excess could lead to adverse outcomes.

### Immunotherapy

TAI is regarded as an epiphenomenon of generalized immune dysfunction; therefore, immunotherapy arouses interest, especially since progressively more evidence indicates that levothyroxine has failed to improve fertility and pregnancy outcomes. Unfortunately, randomized studies using oral steroids (small doses of prednisolone) are scarce. The results of two small RCTs (107, 108) showed improved pregnancy rates in TAI women undergoing ART, but the miscarriage rate was still high (up to 75%). The experience of infertility clinics with intravenous immunoglobulin (IVIG) use in the general population of women with recurrent miscarriage is limited, and evidence for the effect of IVIG in recurrent pregnancy loss is weak. Two recent meta-analyses of IVIG use in recurrent pregnancy loss found no evidence of improved live birth rate (109, 110). However, a higher live birth rate was demonstrated in some publications, as well as beneficial changes in immune profile (*i*.*e*., a decrease in Th1/Th2 ratio and NK cells) (111, 112). Only a few observational studies concerning IVIG use in TAI women with recurrent miscarriage have shown a successful live birth rate of 80% to 90% (113, 114). Further well-designed RCTs are needed to establish the true efficacy of IVIG. In addition, high costs and possible side effects, including anaphylactic reactions and the risk of thrombosis, must be taken into consideration.

In **Table 2**, the current recommendations of endocrine societies (12, 78, 115) for the clinical care of women with TAI who are pregnant or who are planning pregnancy are presented.

**Table 2:** Recommendations regarding the treatment for thyroid autoimmunity during preconception period and pregnancy.

ASRM, 2015 (115)	ATA, 2017 (12)	ETA, 2021 (78)
Preconception		
Not discussed	For women attempting natural conception: no recommendations for treatment with LT4, selenium, and glucocorticoids	For women undergoing ART: No recommendations for routine LT4 treatment in euthyroid women with TAI before ART Consider LT4 treatment with a low dose of 25–50 μg/day before ovarian stimulation in women with TAI and TSH levels >2.5 and <4.0 mIU/L/ULRR in the following cases: • ovarian causes of subfertility • age >35 years • history of recurrent miscarriage • high levels of thyroid antibodies
	For women undergoing ART: LT4 may be considered in a low starting dose (25–50 µg/day) Glucocorticoid therapy not recommended	
Pregnancy		
Consider treatment with LT4 in TPOAb-positive women when TSH >2.5 mIU/L	Consider treatment with LT4 in a low starting dose (25-50 μg/day): • when TPOAb-positive and 2.5 mIU/L < TSH <pregnancy-specific reference range (or 4 mIU/L) • in women with recurrent pregnancy loss	Not discussed
	LT4 not recommended in the prevention of premature delivery	
	Intravenous immunoglobulin treatment not recommended in women with recurrent pregnancy loss	
	Selenium not recommended in TPOAb-positive pregnant women	
	TPOAb- or TgAb-positive euthyroid pregnant women should be monitored with TSH measurement every 4 weeks until midgestation and at least once near 30 weeks of gestation	

ASRM, American Society for Reproductive Medicine; ATA, American Thyroid Association; ETA, European Thyroid Association; TPOAb, thyroperoxidase antibody; TgAb, thyroglobulin antibody; TSH, thyroid-stimulating hormone; LT4, levothyroxine; ART, assisted reproductive technology; TAI, thyroid autoimmunity; ULRR, upper limit of the reference range.

## Conclusions

Thyroid autoimmunity is not only connected with a possible thyroid hormone deficiency but often represents a broader spectrum of immune disturbances that lead to decreased fertility and an increased risk of pregnancy loss. A better understanding of the pathophysiological pathways of TAI is the cornerstone for successful therapies in the future. Levothyroxine supplementation appeared to be ineffective in preventing adverse pregnancy outcomes, so future research should probably be directed toward repairing the immune imbalance.

## Author contributions

KT: conception and study design, literature review, and preparing the paper. MG-C: conception, literature review, and preparing the manuscript. PG and JK: preparing and critical revision of the manuscript. All authors contributed to the article and approved the submitted version.
